# The Cell Cycle Ontology: an application ontology for the representation and integrated analysis of the cell cycle process

**DOI:** 10.1186/gb-2009-10-5-r58

**Published:** 2009-05-29

**Authors:** Erick Antezana, Mikel Egaña, Ward Blondé, Aitzol Illarramendi, Iñaki Bilbao, Bernard De Baets, Robert Stevens, Vladimir Mironov, Martin Kuiper

**Affiliations:** 1 Department of Plant Systems Biology, VIB, Technologiepark 927, B-9052 Gent, Belgium; 2 Department of Molecular Genetics, Ghent University, Technologiepark 927, B-9052 Gent, Belgium; 3 School of Computer Science, University of Manchester, Oxford Road, Manchester M13 9PL, UK; 4 Department of Applied Mathematics, Biometrics and Computer Science, Ghent University, Coupure links 653, B-9000 Gent, Belgium; 5 Noray Bioinformatics, SL Parque Tecnológico 801 A, 2°, 48160 Derio (Bizkaia), Spain; 6 Department of Biology, Norwegian University of Science and Technology, Høgskoleringen 5, NO-7491 Trondheim, Norway

## Abstract

The Cell Cycle Ontology ( is an application ontology that automatically captures and integrates detailed knowledge on the cell cycle process. Cell Cycle Ontology is enabled by semantic web technologies, and is accessible via the web for browsing, visualizing, advanced querying, and computational reasoning. Cell Cycle Ontology facilitates a detailed analysis of cell cycle-related molecular network components. Through querying and automated reasoning, it may provide new hypotheses to help steer a systems biology approach to biological network building.

## Abstract 2

A software resource for the analysis of cell cycle related molecular networks.

## Rationale

Molecular biology has spent the past two decades cataloguing genes, expression levels, proteins, molecular interactions and more. The combination of all these catalogues should enable a biologist to start building a comprehensive picture of a biological system rather than only looking at the individual components. The formation of representations of these components into a network that describes a biological system constitutes the first step in allowing a biologist to develop an understanding of the behavior of a system. If adequate kinetic and other parameters can be obtained or estimated, such models can be used for network simulations in a mathematical framework, making them particularly useful to study the emergent properties of such a system [1-5]. These models provide the basis for much of systems biology that is built on integrative data analysis and mathematical modeling [6-9]. In systems biology, dynamic simulations with a model of a biological process serve as a means to validate the model's architecture and parameters, and to provide hypotheses for new experiments.

Complementary to such model-dependent hypothesis generation, the field of computational reasoning promises to provide a powerful additional source of new hypotheses concerning biological network components. The integration of biological knowledge from various sources and the alignment of their representations into one common representation are recognized as critical steps toward hypothesis building [10,11]. Such an integrated information resource is essential for exploration and exploitation by both humans and computers, as in the case of computers via automated reasoning [12].

### Bio-ontologies

While it is easy to compare nucleic acid or polypeptide sequences from different bioinformatics resources, the biological knowledge contained in these resources is very difficult to compare as it is represented in a wide variety of lexical forms [13-15], and there are no tools that facilitate an easy comparison and integration of knowledge in this form. This is where ontologies can provide assistance.

Ontologies represent knowledge about a specific scientific domain, and support a consistent and unambiguous representation of entities within that domain. This knowledge can be integrated into a single model that holds these domain entities and their term labels, as well as their connecting relationships [16]. A well-known example of such an ontology is the Gene Ontology (GO) [17]. Therefore, an ontology links term labels to their interpretations, that is, specifications of their meanings, defined as a set of properties.

Ontologies not only provide the foundation for knowledge integration, but also the basis for advanced computational reasoning to validate hypotheses and make implicit knowledge explicit [18,19]. Integrated knowledge founded on well-defined semantics provides a framework to enable computers to conceptually handle knowledge in a manner comparable to the handling of numerical data: it allows a computer to process expressed facts, look for patterns and make inferences, thereby extending human thinking about complex information. On a more technical level, computational reasoning services can also be used to check the consistency of such integrated knowledge, to re-engineer the design of parts of the entire ontology or to design entirely new extensions that comply with current knowledge [20].

Generally speaking, ontologies that model domain knowledge are developed through an iterative process of refinement, an approach common in the field of software engineering [21]. Ontology development has been pursued for many years, and while several methodologies have been proposed [22-29], none has been widely accepted. The Open Biomedical Ontology (OBO) project [30], however, aims to coordinate the development of bio-ontologies (for example, the GO and the Relation Ontology (RO) [31], among many others). The OBO foundry [32] has provided a set of principles to guide the development of ontologies. These ontologies have gained wide acceptance within the biomedical community [33] as a means for data annotation and integration and as a reference.

Biological information is known to be difficult to integrate and analyze [34]. One of the reasons for this is that biologists are inclined to invent new names and expressions for, for example, proteins and their functions that others have already named. This has led to high incidences of synonymy, homonymy and polysemy that plague biomedicine. Furthermore, biological knowledge is often not crisp, as evidenced by the widespread use of quantifiers such as 'often', 'usually' and 'sometimes'. Finally, the sheer volume and complexity of biological data and the diversity of representational formats provide profound challenges for efficient biomedical knowledge management. Altogether, this calls for a concerted effort of experts from the biomedical and computational sciences to organize and facilitate the integration and exploitation of rapidly accumulating biological information.

### Application ontologies in the life sciences and their role in systems biology

**Figure 1:**
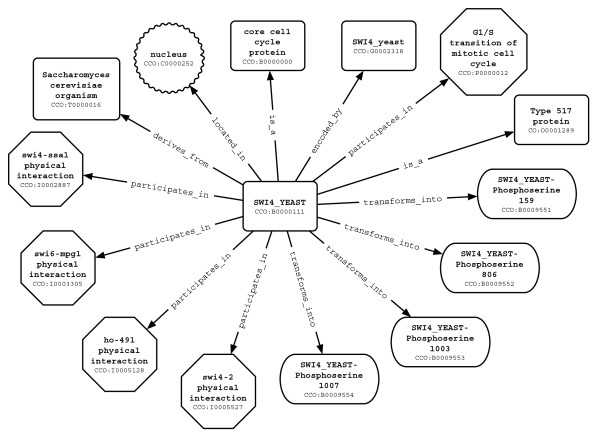
Example of the local neighborhood of the protein SWI4_YEAST: some of the types of relationships used within CCO depict how a given protein (SWI4_YEAST) is connected to the organism it belongs to (*S. cerevisiae*), its coding gene (SWI4_yeast), biological processes (G1/S transition of mitotic cell cycle), cellular localization (nucleus), interactions (physical interactions), protein transformations (post-translational modifications), and its orthology group.

Application ontologies define relevant concepts for a particular application or use [35]. They can be built by combining domain ontologies (or parts of domain ontologies) or serving as 'a reference', and they can be extended according to the needs of a particular application. Application ontologies are intended to be directly embedded into knowledge bases on which different applications can be run, such as data mining and hypothesis generation. Application ontologies can play an important role in exploiting the formalization of domain knowledge, thereby facilitating the integration of different types of information (for example, knowledge about biological processes and subcellular localizations, both parts of GO). Figure 1 shows a sample piece of knowledge composed of such integrated information. This schematic representation gives a minimal but context-linked notion of a specific protein and its environment of functional characteristics (for example, where it is located, in which processes it participates, and by which gene it is encoded).

A successful application ontology may form the core of an efficient and effective management system. Such a system combines data extraction methods, data format conversions and a variety of information sources. To illustrate the potential use of application ontologies for the life sciences, we have designed and built a knowledge management system that facilitates the analysis of cell cycle control.

### Why focus on the cell cycle process?

The eukaryotic cell cycle, or cell division cycle, is the series of events that happen between two consecutive cell divisions that underlie cell multiplication. The molecular events that control the cell cycle are ordered and directional; that is, each process occurs in a sequential fashion and it is impossible to reverse the cycle. The cell cycle control network is complex and is thought to include hundreds of proteins [36,37].

Although the basic principles of cell cycle control are now well documented [38], we are far from having a complete understanding of all the intricacies of the underlying system. A deeper knowledge of the cell cycle control system is essential to the understanding of the growth and development of eukaryotic organisms. In turn, this is necessary in order to be able to combat numerous diseases in which cell cycle aberrations are involved, such as cancer.

Part of this knowledge has already been incorporated into dynamic system models that are being exploited to test, refine and generate hypothesis [39]. This holistic and integrative approach in biological research, also called systems biology, is gaining momentum [40,41] and is leading to novel insights into cell machinery [37,42,43]. To further augment the cell cycle research with computational approaches, we have built the Cell Cycle Ontology (CCO), which integrates a wide variety of knowledge sources pertinent to the cell cycle.

## Results and discussion

### The Cell Cycle Ontology application ontology

CCO is built to provide laboratory biologists with a one-stop shop for cell cycle knowledge and to have access to an integrated knowledge system that can be used to explore the potential power of automated reasoning. CCO comprises information from a number of resources that contain relevant information about the cell cycle process, such as GO [44], RO, the IntAct database [45], the National Center for Biotechnology Information (NCBI) taxonomy [46], the UniProt knowledge base [47], and putative orthology relationships derived with the OrthoMCL clustering algorithm [48,49]. All the information is integrated into a single framework that is supported by the ontologies. The integrated knowledge system supports queries that are not feasible with the original, individual and separate information sources.

Bio-ontologies and their presentations have been made accessible through existing software tools (such as OBO-Edit [50], Protégé [51]), or web-based tools such as BioPortal [52], which can be used to create new terms and relationships and to explore and analyze these ontologies). The most frequently used biomedical ontologies are provided in the Open Biomedical Ontology format (OBOF) [53], while some are also natively available in the Web Ontology Language (OWL) [54] (though the OBOF can be transformed into an OWL representation [55-58]). OWL provides a means of creating semantically rich ontologies with ample possibilities for querying and computational reasoning. Therefore, we converted the wealth of information available in the OBOF, and the highly curated information from public data sources, into the more expressive OWL representation in order to exploit richer forms of computational reasoning.

CCO is extensible, and the CCO integration architecture can accommodate additional ontologies if necessary. In addition, a broad range of export formats from CCO (in particular, OWL and Resource Description Framework (RDF)) enables virtual integration with external sources (controlled vocabularies translated into RDF such as Medical Subject Headings (MeSH) [59]), allowing for queries that address these disparate resources through Semantic Web technologies [60,61].

### Knowledge representation in the Cell Cycle Ontology

CCO is a resource that can directly support systems biology. Systems biology is essentially a model-driven approach to biological research, in which a model of a biological process serves to integrate all the available information (network components and their interactions). A model simulation allows for an understanding of network behavior, including changes to the entities, describing these changes in terms of what these entities are, where they are located and when these statements hold. To this end, the knowledge of entities and their interactions needs to be represented in a mathematical framework that facilitates dynamic simulations.

Similarly, to computationally reason about temporal and spatial aspects of a biological process, this knowledge should be represented by a semantically rich and strict language (for example, OWL) to exploit computational reasoning tools. Automated reasoners for OWL do not directly support either temporal or spatial reasoning. It is possible, however, to make representations of temporal and spatial aspects of knowledge and then reason about them in a way that is adequate for many application settings.

Within cell cycle related research, a scientist may be interested in a particular protein (what) for which the localization (where) and specific phase of the cell cycle (when) are important analysis components. To represent the linkage between all these different terms, CCO uses relationships as follows. Let: B be a protein; C be a cellular location in which B might be present; G be the gene that codes for B; P be a biological process in which B participates; I be an interaction in which B takes part; and T be the organism that is the source of B.

These relationships provide the basis for the atomic elements of knowledge about the protein B: 'B located in C', 'B coded by G', 'B participates in P', 'B participates in I', and 'B has source T'. The existing relationships also have an inverse relationship such as 'P has participant B', 'G codes for B', 'C location of B', 'T source of B'. An example is shown in Figure 1.

### Cell Cycle Ontology contents

CCO supports four model organisms: *Homo sapiens*, *Saccharomyces cerevisiae*, *Schizosaccharomyces pombe*, and *Arabidopsis thaliana*. There is an individual ontology for each of the supported organisms. There is also an integrated ontology that additionally contains (putative) orthology relationships obtained through OrthoMCL clustering. Currently, the integrated CCO contains 132,263 terms: 90,643 proteins (including their modified forms), 21,039 genes and 20,581 protein-protein interactions, and it further comprises 30 types of relationships (properties) (see Tables 1, 2 and 3 for detailed information). The contents of CCO can be viewed and analyzed through a wide variety of tools (see below).

**Table 1:** Organism-specific ontology figures

	Ontology				
Entity	At	Hs	Sc	Sp	Total
Proteins	3,572	26,220	14,685	2,388	46,865
Genes	3,027	8,699	4,498	1,439	17,663
Protein protein interactions	1,524	8,707	9,903	447	20,581

The numbers shown are of some important entities presently contained in CCO (for example, cell cycle genes) for each of the organism-specific ontologies (*A. thaliana *ontology (At), *H. sapiens *ontology (Hs), *S. cerevisiae *ontology (Sc) and *S. pombe *ontology (Sp)).

**Table 2:** CCO protein figures

	Ontology				
	
Type of proteins	At	Hs	Sc	Sp	Total
Core cell cycle	3,276	9,114	1,648	1,348	15,386
Added from IntAct	166	1,671	2,777	80	4,694
Modified proteins added from UniProt	126	15,328	10,200	926	26,580
					

Total	3,572	26,220	14,685	2,388	46,865

This table shows the number of cell cycle related proteins that were integrated into the four species-specific ontologies for the model organisms: *A. thaliana *(At), *H. sapiens *(Hs), *S. cerevisiae *(Sc) and *S. pombe *(Sp). See 'Data integration' in Materials and methods for the definition of the term 'core cell cycle protein'.

**Table 3:** Integrated ontology figures

	Ontology				
	
Entity	At	Hs	Sc	Sp	Total
Proteins	14,892	54,109	18,007	3,635	90,643
Genes	4,595	10,005	4,695	1,744	21,039
Orthology types	-	-	-	-	5,772

Figures are shown for the composite ontology (CCO): union of the four organism-specific ontologies (*A. thaliana *(At), *H. sapiens *(Hs), *S. cerevisiae *(Sc) and *S. pombe *(Sp)) plus their orthology relationships. The OrthoMCL execution adds 5,772 clusters containing at least one core cell cycle protein (see 'Data integration' in Materials and methods for the definition of the term 'core cell cycle protein') together with their proteins to CCO; the total number of proteins in CCO is 90,643. Numbers are given for some of the main entities (for example, cell cycle proteins) in the composite ontology (CCO).

### Main features of the Cell Cycle Ontology

CCO is protein centric, meaning that proteins are used as 'hubs' to integrate and connect knowledge. The semantic integration of knowledge creates synergy by allowing queries that would not otherwise be possible. For example, OBO ontologies can be queried by tools such as OBO-Edit [62], the OBO Explorer [58] and AmiGO [63], but none of these can deal with a query such as 'return the orthologs of a protein X and include all the biological processes and molecular functions in which these orthologs participate'. Due to our integrative approach and selection of information sources, CCO is an information-rich ontology that offers many advantages for cell cycle researchers. The main characteristics and functionalities of CCO, described in more detail below, can best be summarized as follows: integrated turnkey system - CCO evolves toward a one-stop shop for cell cycle researchers; exploratory analysis - CCO provides ample possibilities for browsing, visualizing and searching; querying facilities - CCO offers advanced methods to retrieve data; reasoning exploitation - the integrated knowledge is structured to allow for classification, consistency checking, and more advanced implementations that may provide new hypotheses.

CCO has been made available in a wide range of formats to accommodate a suite of popular visualization and analysis tools, ensuring maximum flexibility of interaction with the ontology: OBOF, OWL [64], RDF [65], the eXtensible Markup Language (XML) [66], DOT [67] and the Graph Modeling Language (GML) [68]. Those formats can be classified into three groups according to the way the user interacts with CCO: a basic exploration of the structure (OBOF), expressive queries including the possibility of combining CCO with other resources (XML, RDF and OWL), and visual exploration (GML, XML - visANT [69] - and DOT). The representations are described in detail as follows.

OBOF is the *de facto *standard for knowledge representation in the bio-ontology community. Many tools have been built to accommodate OBOF (for example, OBO-Edit [50] and OBO Explorer [58]), and are widely used by biologists. Much of the biological knowledge already captured in ontologies is represented in OBOF [70]. This is why we chose the OBOF resource as the starting point for the CCO pipeline. The OBOF version of CCO is compliant with version 1.2 of the OBOF specification. OBOF, however, offers little in the way of native reasoning services and even lacks a semantic infrastructure for knowledge integration, such as RDF and OWL do via Uniform Resource Identifiers (URIs). OBOF queries are limited to simple exploration of the ontology structure.

**Figure 2:**
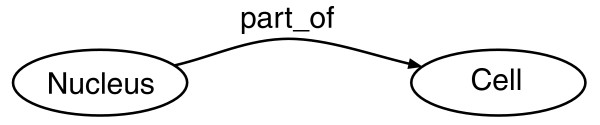
Simple RDF triple sample showing the subject (Nucleus), the predicate (part_of) and the object (Cell).

**Figure 3:**
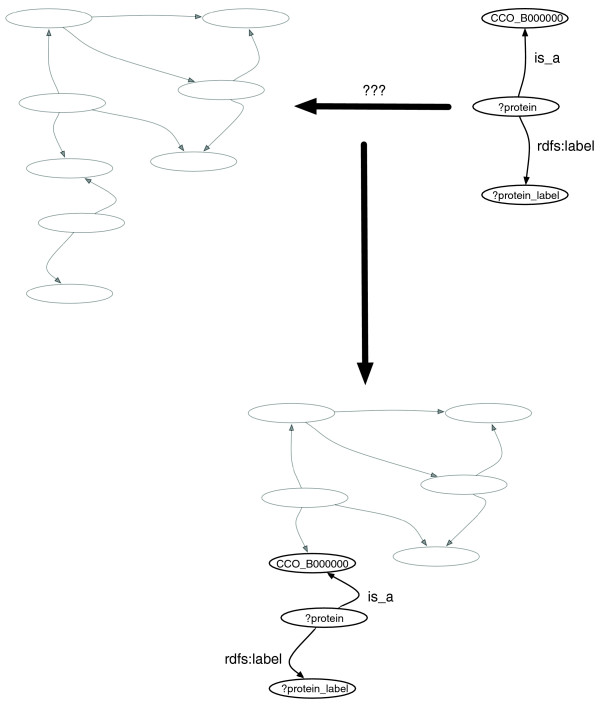
RDF matching model: while querying an RDF model, a matching process is performed against the graph model. In the sample, the triples '?protein is_a CCO_B0000000' and '?protein rdfs:label ?protein label' are matched against the graph on the left.

An RDF model is a collection of triple patterns, also simply named 'triples', comprising a subject, a predicate and an object (Figure 2) connected to each other in a graph (for example, the subject of one triple can be the object of another triple). An RDF graph can be flexibly and efficiently queried with the graph query language SPARQL [71] (Figure 3). We have loaded the RDF version of CCO into Open Virtuoso [72] to enable complex queries via SPARQL. In addition, a SPARQL query form [73] and a SPARQL query service [74] are also available to exploit CCO. The CCO RDF allows for a first step toward exploiting Semantic Web technologies [75] as it offers the possibility to integrate knowledge from external resources [76]. Tools such as RDFScape [77] (a plug-in for Cytoscape [78]) can also be used to explore this CCO representation.

The OWL version of CCO is the most expressive one and exceeds the other versions in information content as new axioms (see Materials and methods) have been added to exploit its language capabilities (the other versions are equivalent in content to the original ontologies in OBOF). OWL also allows integration of other ontologies within CCO by using an importing mechanism based on URIs, meaning that extant encoded knowledge from other resources can be effectively added and exploited. Ontologies expressed in OWL, however, often cause performance limitations to the extent that it is prohibitive for specific tools, such as Protégé, when launching very complex queries. OWL reasoners (Pellet [79], FaCT++ [80], RACERPro [81], and KAON2 [82]) can have problems in dealing with large ontologies (such as CCO) and sometimes fail without explanation [83]. Additionally, the OWLDoc server [84] allows online queries over CCO [85].

XML allows efficient data processing and programmatic access to the ontology. XML has less expressivity than RDF or OWL in terms of semantics. The structured document enabled in XML also supports querying (for example, with technologies such as XQuery [86]).

GML, XML (visANT) and DOT allow visual exploration of CCO by tools such as Cytoscape [78], visANT and Graphviz [87]. In particular, visANT provides a very user-friendly way to examine the CCO network of terms and relationships.

### Querying the Cell Cycle Ontology with SPARQL

The SPARQL syntax is based on the triple pattern of RDF and, therefore, allows for a detailed specification of a small graph pattern, thus a collection of interconnected triples, for which the graph should be queried. When performing a query with SPARQL, a small RDF graph pattern is built in which any of the elements of any triple can be a variable (variable names are prepended in the query with the sign ? or $). This query pattern is used to match against the complete RDF graph and any matching structure (collection of triples) is retrieved (Figure 3).

A query can also specify which variables in the query pattern should be shown in the answer. One of SPARQL's strengths is its ability to specify various target graphs that could be used in the same query, resulting in their subsequent combination and effectively constituting an efficient data integration mechanism. As the pointers to the graphs are URIs, knowledge represented in dispersed RDF resources can be combined in a powerful way.

In order to design SPARQL queries on CCO, it is sometimes necessary to deal with CCO identifiers. The following query shows how to retrieve a term name (called 'label' in RDF) corresponding to a given CCO identifier ('CCO_B0000000' in this example). First, a base URL is defined (BASE), and then the prefixes (PREFIX) are set to avoid the repetition of long parts of URIs in the queries. The variables (columns) to be shown in the solution are specified in the SELECT statement. Finally, the query pattern is defined in the WHERE block. The specification of the graphs that should be used (for example, 'cco') is considered as a part of the query pattern. The results table will display the term label: 'core cell cycle protein' (see 'Data integration' in Materials and methods for the definition of 'core cell cycle protein').

BASE <>

PREFIX rdfs:<>

PREFIX ssb:<>

SELECT ?term_label

WHERE {

   GRAPH <cco> {

      ssb:CCO_B0000000 rdfs:label ?term_label

   }

}

A similar query can be employed to retrieve a CCO identifier using a term label. The following query retrieves the CCO identifier ('CCO_B0002337') of the protein with the label 'WEE1_ARATH':

BASE <>

PREFIX rdfs:<>

SELECT ?unique_id

WHERE {

   GRAPH <cco> {

      ?unique_id rdfs:label 'WEE1_ARATH'@en

   }

}

More sophisticated searches based on regular expressions can also be performed as illustrated in the following query that retrieves all the terms having the keyword 'p53' anywhere within the label (the flag 'i' enables case-insensitive expression lookups):

BASE <>

PREFIX rdfs:<>

SELECT ?unique_id ?name

WHERE {

   GRAPH <cco> {

      ?unique_id rdfs:label ?name.

      FILTER regex(str(?name), 'p53','i')

   }

}

Consider the simple query 'retrieve the names (labels) of all core cell cycle proteins from *S. pombe*'. These are the proteins annotated with cell cycle terms by the Gene Ontology Annotation (GOA) [88] group. The query pattern consists of two triples. The first triple will match any triple that relates any subject through the 'is_a' predicate to the 'CCO_B0000000' object (core cell cycle protein) and the second triple will match any triple whose subject is the same as in the first triple, the variable ?protein (defined by ? or $ in front of a string name), and has the predicate 'rdfs:label' pointing to any object. The result is a column (?protein_label) with the label of 1,359 core cell cycle proteins in *S. pombe *(for example, CDC24_SCHPO). Figure 3 illustrates the query pattern that corresponds with the following SPARQL query:

BASE <>

PREFIX rdfs:<>

PREFIX ssb:<>

SELECT ?protein_label

WHERE {

   GRAPH <cco_S_pombe> {

      ?protein ssb:is_a ssb:CCO_B0000000.

      ?protein rdfs:label ?protein_label

   }

}

The following SPARQL query on the *A. thaliana *graph allows users to infer a putative location for proteins with no documented cellular locations. The assumption behind such a query is that two proteins that participate in the same interaction are likely to share the same cellular location, for example, the 'nucleus' (CCO_C0000252):

BASE <>

PREFIX rdfs:<>

PREFIX ssb:<>

SELECT

   ?prot_in_the_nucleus

   ?prot_to_study

   ?interaction_label

WHERE {

   GRAPH <cco_A_thaliana> {

      ?interaction a ssb:interaction.

      ?interaction rdfs:label ?interaction_label.

      ?prot_A ssb:participates_in ?interaction.

      ?prot_B ssb:participates_in ?interaction.

      ?prot_A rdfs:label ?prot_in_the_nucleus.

      ?prot_B rdfs:label ?prot_to_study.

      ?prot_A ssb:located_in ssb:CCO_C0000252.

      OPTIONAL {

         ?prot_B ssb:located_in ?location_B.

      }

      FILTER (!bound(?location_B))

   }

}

The query returns 48 proteins (for example, DMC1_ARATH, SEM12_ARATH) having an interaction with a documented nuclear protein, meaning their own cellular location is also likely to include 'nucleus' at some point. These results and, more generally, any answer to a query on CCO simply reflects the information in the original sources, but their integration enables the construction of new hypotheses. For some questions, the integrated CCO graph must be used. For instance, to retrieve the orthologs of the protein TIP41_YEAST from *S. cerevisiae *(CCO_B0001243) and the processes in which these orthologs participate, the following query can be used:

BASE <>

PREFIX rdfs:<>

PREFIX ssb:<>

SELECT

   ?prot_label

   ?biological_process_label

WHERE {

   GRAPH <cco> {

      ssb:CCO_B0001243 ssb:is_a ?ortholog_cluster_protein.

      ?prot ssb:is_a ?ortholog_cluster_protein.

      ?prot rdfs:label ?prot_label.

      ?ortholog_cluster_protein rdf:type ssb:type_protein.

   OPTIONAL {

      ?prot ssb:participates_in ?biological_process.

      ?biological_process rdfs:label ?biological_process_label

   }

   FILTER(?prot != ssb:CCO_B0001243)

   }

}

The query returns 63 distinct putative orthologs, of which 55 are not documented to participate in any known process. Thus, with this result these proteins can be hypothesized to participate in the same process as 'TIP41_SCHPO'. To retrieve the identity of the processes in which 'TIP41_SCHPO' participates, a new query must be built that returns the answer 'G2/M transition of mitotic cell cycle':

BASE <>

PREFIX rdfs:<>

PREFIX ssb:<>

SELECT ?process_label

WHERE {

   GRAPH <cco> {

      ssb:CCO_B0001243 ssb:participates_in ?process.

      ?process rdfs:label ?process_label

   }

}

More examples of biological queries can be found at [73].

Finally, we used SPARQL to analyze the subcellular distribution of cell cycle proteins. For that, we used the core cell cycle proteins subset of the CCO. First, we analyzed the distribution among the three major cellular compartments - the cytoplasm, nucleus and cell membrane. We found that the majority of cell cycle proteins are located in the nucleus (755) and the cytoplasm (356), where the majority of cell cycle events are known to take place [38]. Twenty-five cell cycle proteins were found to be located in the cell membrane. These are likely to play a role in signaling to the cell cycle machinery.

We looked in more detail at the distribution of cell cycle proteins in the cytoplasm. As expected, the majority of cell cycle proteins are found in the cytosol (280). We also wanted to see if there were cell cycle proteins in the membrane bounded organelles other than in the nucleus. To our surprise, all of the analyzed organelles contained cell cycle proteins: the endoplasmic reticulum (46), the Golgi apparatus (19) and the mitochondrion (43). One could hypothesize that the cell cycle proteins located in the first two compartments are involved in the build-up of a new cell membrane and cell wall between the two daughter cells. It is much more difficult, however, to envision how mitochondrial proteins could be involved in the cell cycle. Even more strikingly, six mitochondrial proteins were found to play a role in the regulation of the cell cycle. Provided the cellular compartment annotations are correct, and if taken up by cell cycle researchers, these results may possibly lead to the discovery of novel mechanisms of cell cycle regulation.

An alternative hypothesis to explain a cell cycle role for proteins known to be located in membrane bounded organelles other than the nucleus is to suggest that these proteins are also present outside of those organelles. For example, if a protein can be located in both the mitochondrion and the cytosol, then the cell cycle function of the protein can be exerted in the cytosol, but not in the mitochondrion where it may fulfill a different role. Therefore, we analyzed alternative locations of the proteins in question. We identified 9, 5 and 15 core cell cycle proteins from the endoplasmic reticulum, Golgi apparatus and mitochondrion, respectively, that have additionally cytosolic or nuclear localization. These proteins have an unusual combination of locations, and merit further investigation with respect to the molecular mechanisms underlying their ability to be localized to apparently incompatible locations. This also highlights the need to indicate when and where functions assigned to a protein are valid.

### Automated reasoning over bio-ontologies

#### Description logics and automated reasoners

Description Logics (DL) [89] and Semantic Web technologies [60,61] provide a foundation for the management and exploitation of knowledge in ontologies. The type of OWL used for CCO is based on DL, which is a family of logic-based knowledge representation formalisms that describe a domain in terms of concepts (classes), roles (properties or relationships) and individuals (instances). OWL-DL offers an optimal trade-off between expressivity and computational tractability [89]. OWL-DL can be considered to be sufficiently expressive in order to represent a wide variety of biomedical knowledge [90], while it offers support for automated reasoning. It has become one of the standard languages for representing ontologies in the semantically strict form that supports automated reasoning.

DL reasoners are computational tools to: ensure that an ontology does not contain any contradictory facts (consistency checking); compute the subclass relation between each named class to create the class hierarchy (classification); find the most specific classes to which an individual belongs (realization); and retrieve information from an ontology (querying).

Ontology curators can use DL reasoners to minimize the term redundancy, while maintaining sufficiently detailed descriptions and consistency of the contents [18,19]. Moreover, reasoning tools can also be used to find new classes (either more specific or general) [20]. Finally, and in this context most importantly, reasoning tools can also be used in biological research for information retrieval and the generation of new hypotheses that are consistent with the knowledge captured in the ontology.

#### Representing biological knowledge with OWL

OWL-DL queries can be more fine-grained than RDF queries since the semantic model of OWL-DL allows more expressivity. The OWL semantics is based on sets (classes) of instances (individuals). Classes can be subclasses of other classes, if and only if all the instances of the subclass are also instances of the superclass, although the superclass has other instances that do not belong to the subclass. For example, in GO the well-known 'is a' hierarchy is founded on this concept.

**Figure 4:**
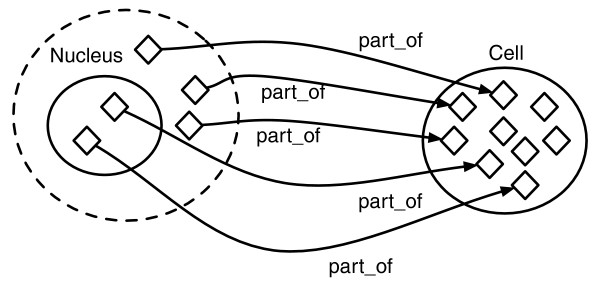
OWL property (part_of) sample: the property 'part of' links individuals belonging to a class (for example, 'Nucleus') to individuals of the class 'Cell'. A restriction of the type 'some part_of Cell' on the class 'Nucleus' defines an anonymous class (dotted shape), and will imply that individuals belonging to the class 'Nucleus' also belong to (are 'part_of') the class 'Cell'.

Relationships in OWL-DL are interpreted as existing between pairs of individuals. Restrictions on classes define which and how many relationships the instances of that class must hold. When a restriction is defined, an anonymous class is defined (Figure 4, dotted shape), and the class to which the restriction is added becomes a subclass or equivalent class of that anonymous class. For instance, the restriction 'subClassOf part of some Cell' in the class 'Nucleus' states that every instance of the class 'Nucleus' must have at least one relationship along the property 'part_of' to an instance of the class 'Cell' (other quantifiers can be used in these restrictions such as 'only', 'min', 'max' and 'value', and Boolean operators such as 'and', 'or', and 'not').

If the restriction is added as a superclass of the class that is being defined (the class being defined is a subclass of the restriction, as in the example above), the restriction is known as a 'necessary condition'. A necessary condition is a condition that all the instances of the class must fulfill, but is not enough in itself to define class membership. Therefore, if an instance is found that has at least one 'part_of' relationship to 'Cell', it does not mean that it is a member of the class 'Nucleus'. If a restriction is added as an equivalent class of the class that is being described, then this restriction is known as a 'necessary and sufficient' condition (if an instance with at least one 'part of Cell' relationship is found, it is a member of the class 'Nucleus'). Restrictions can also be composed and nested such as 'part of some (participates in only (mitosis or meiosis))', providing a powerful mechanism for expressing complex conditions.

OWL works under the Open World Assumption (OWA), in which something not known to be true is not assumed to be false but merely to be 'unknown'. This is in contrast to database management systems that work with the Closed World Assumption. In database management systems, anything that is not demonstrated by the stored data to be true is assumed to be false. As an example, let us assume that in a system the following and only the following is stated: 'protein CDC25_YEAST participates in the regulation of the cell cycle process', and if the query: 'does protein CDC25_YEAST participate in the regulation of spindle elongation?' is launched, the answer will depend on the type of system. In a Closed World Assumption-based system (for example, a typical database management system), the answer will be 'no', whereas in an OWA-based system (for example, an OWL knowledge base) the answer will be 'unknown'; in other words, protein CDC25 YEAST might also participate in other processes, and will remain 'unknown' until it is explicitly stated that it either does or does not participate in that process. The OWA model complies well with the Semantic Web vision, in which knowledge is continuously evolving, and efficiently accommodates domains with incomplete knowledge such as the cell cycle process.

In an OWL ontology, the knowledge is asserted by the user, and the reasoner makes the axioms that are implicit in such asserted knowledge explicit, therefore inferring axioms that flag unnoticed items of knowledge. Therefore, the complexity of OWL queries depends on the complexity of the asserted and inferred knowledge present on the ontology. An OWL query can be regarded as an 'anonymous class', and, therefore, the user may ask the reasoner for different answers (for example, retrieve superclasses, ancestor classes, equivalent classes, subclasses, descendant classes or instances of the anonymous class). CCO provides an attractive starting point to exploit all these querying possibilities by enriching it with particular/customized axioms. These axioms could be limited to subsets of CCO that will enable focusing on particular aspects of cell cycle research such as endoreduplication. The examples described below only show some of the added value that could be gained from having an ontology expressed in OWL.

#### Examples of automated reasoning in the Cell Cycle Ontology

Consider the simple query: 'Which cell cycle related proteins participate in a reported interaction?' In the Manchester OWL syntax [91], that query would be:

   protein and

   participates_in some 'interaction type'

where the classes 'protein' and 'interaction type' have CCO_U0000005 and CCO_Y0000001, respectively, as their CCO identifiers. When a query is launched, an anonymous class is built on the fly. The extension of that anonymous class is the set of individuals that are members of the class 'protein' with at least one relationship (due to the 'some' keyword) along the property 'participates_in' to an individual of the class 'interaction type'. The expressions to create an anonymous class can be of arbitrary complexity: combination of constructors, nested expressions, combinations of different types of restrictions, and so on. For example, if we want to know which proteins (CCO_U0000005) participate in 'mitosis' (CCO_P0000081) or any process that is a part of it we may use the expression below, which shows the versatility and power of OWL expressions:

   protein and

   participates_in some (

      mitosis or part_of some mitosis

   )

The extension of this anonymous class is the set of instances that are members of the class 'protein' (CCO_U0000005) and have at least one relationship along 'participates in' to an individual of the class 'mitosis' (CCO_P0000081) or to an individual that has at least one relationship along 'part_of' to an individual of 'mitosis'. Reasoning is used to exploit the transitivity of the 'part_of' and subsumption relationships. Thus, a process that is not directly part of mitosis, but that is part of a process that is part of mitosis, will be taken into account, even though such knowledge is not asserted in the ontology: such knowledge is inferred by the reasoner, and it is implicit (asserted) in the axioms of the ontology. Another example is retrieving proteins that have at least one interaction with other proteins, and can be located in the cytoplasm as well as in the nucleus:

protein and

participates_in some interaction and

((located_in some (part_of some cytoplasm)) or (located_in some cytoplasm)) and ((located_in some (part_of some nucleus)) or (located_in some nucleus))

This query defines an anonymous class formed by 'proteins' (CCO_U0000005) that participate in at least one 'interaction' (CCO_U0000007), and are located in the 'cytoplasm' (CCO_C0000323) or any part of it, and the 'nucleus' (CCO_C0000251) or any part of it.

As another example, let us consider the query that defines an anonymous class formed by the entities that are the location of proteins participating in the 'S phase' (CCO_P0000014) or any process that is a part of it. This query returns 13 proteins for *H. sapiens *such as 'microtubule organising center' (CCO_C0000385), which is the location of the protein CHM1A_HUMAN that participates in negative regulation of the S phase of the mitotic cell cycle (part of the S phase in the mitotic cell cycle):

   location_of some (

      participates_in some (

         'S phase' or (part_of some 'S phase')

      )

   )

The same procedure can be applied to any defined query with the OWL-DL query service provided by Protégé 4 and also programmatically via the OWL application programming interface (OWL application programming interface) [92].

The OWL-DL version of CCO constitutes a structured and integrated knowledge framework that may serve as a basis for advanced reasoning approaches. Reasoning services can be applied to CCO at different levels, depending on who interacts with the system (such as a molecular biologist or an ontology specialist [93,94]) to check the consistency of knowledge, to validate cell cycle related hypotheses and to make implicit knowledge explicit.

### Cell Cycle Ontology integrated into a platform for cell cycle research

**Figure 5:**
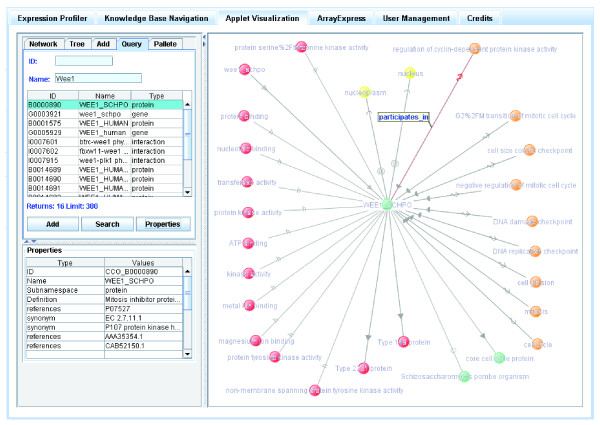
CCO visualization applet. The CCO terms are shown as clickable, color-coded nodes, and mouse actions of the user are translated into pre-defined SPARQL queries that operate on the RDF representation of CCO. The results are returned as an XML file, which is translated into new nodes and edges that are then shown in the display. This visualization sample shows the local neighborhood of the protein WEE1_SCHPO ('Mitosis inhibitor protein kinase wee1').

The querying and analysis of results of SPARQL queries can be intimidating for lay users. We therefore set out to build a visualizer of biological networks specifically for CCO (in the framework of the EU FP6 project DIAMONDS [95]). This visualizer is a JAVA applet that shows a screen consisting of two parts (Figure 5): one section with a panel where applet functionalities are grouped and can be configured, and another panel with a graphical representation of the results of queries (usually in the form of networks) to CCO. SPARQL provides intuitive ways to query hierarchical networks. With a right click of the mouse on any of the nodes shown by the applet, the user can ask for the local neighborhood of a term in the network, for the path to the root and for extra information such as definitions and synonyms. Users can see the networks, personalize them, add or delete elements, change colors, and move and re-design the shape of the networks.

The terms in CCO are basically divided into three types: terms that represent a protein class (for example, 'UBC11_SCHPO'); terms in the upper level ontology (for example, 'biological process'); other ontological terms (for example, 'nucleolus').

The RDF contains explicit 'rdf:type' links from every term node to its type as well as 'rdfs:label' links to their names. All the pre-defined SPARQL queries operate on the URIs of the CCO terms, but they retrieve the 'rdf:type' and 'rdfs:label' for the visual presentation of the results. The types are used for a color coding of the nodes. The links between the terms also have names, defined by the relation types in CCO (for example, interacts with, encoded by, and so on). Some of the relation types, such as the protein interactions, are visualized with special arrows. For more information, please see the DIAMONDS deliverable document D5.4 [96].

### Yet another integrated system?

Efficient use of biological information that, in practice, is widely dispersed strongly relies on its means of access. However, each individual resource mostly informs the researchers about only a part of their biological question. Therefore, life scientists often need to combine several resources in order to gain new insights. Computational systems that provide transparent and integrative access should increase research productivity. Some systems, such as Reactome [97] and PANTHER [98], are examples of developments in that direction. These systems are, however, not so easily expandable to include other domains or information on-the-fly or upon request. More importantly, although those systems provide highly curated information and user-friendly interfaces, the information retrieval is still limited to simple search forms that only allow keyword-based look-ups. The possibility of using the stored information through complex but more specific and informative queries remains limited. Also, these systems lack means to make implicit knowledge explicit; this is where the reasoning services available in CCO offer added value.

CCO adopts a data integration paradigm that can be readily applied to any other domain. The system is readily expandable and can accommodate virtually any other data related to the cell cycle (for example, cell cycle related information from the Kyoto Encyclopedia of Genes and Genomes (KEGG) [99] and Online Mendelian Inheritance in Man (OMIM) [100]). Furthermore, the use of Semantic Web technologies in CCO enables interoperability with other Semantic Web resources and constitutes a step towards a universal, interoperable knowledge architecture [101] for the life sciences. Semantic Web integration alleviates the burden of resource maintenance, allowing for more attention to local improvements (as shown with the reasoning cases in CCO). A universal and interoperable knowledge-based approach can enable the implementation of an effective integrated biology.

Currently, building complex queries over CCO may require some training in semantic technologies. It has always been difficult to develop systems that provide a balanced trade-off between user-friendliness and the possibility of asking complex questions. Nevertheless, it is fair to assume that Semantic Web research will deliver such features in the future. The rich ontology developed to study the cell cycle process highlights the advantages of semantically representing knowledge for further analysis and ontology-driven hypothesis generation. We envision that by improving both content and semantics, the utility of CCO can be considerably increased.

## Materials and methods

### Data integration pipeline

**Figure 6:**
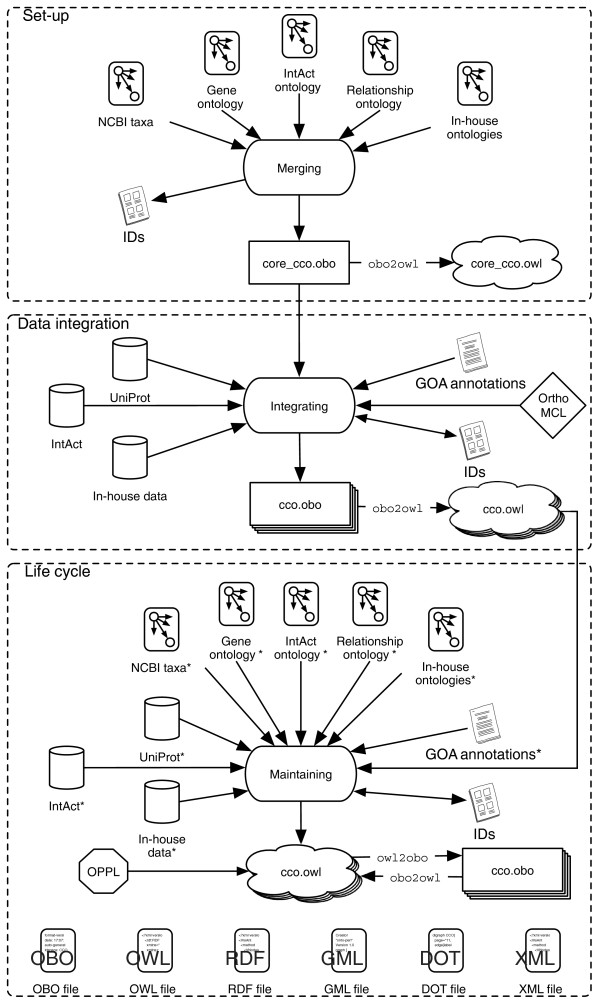
CCO pipeline. The CCO data integration pipeline scheme plots the principal phases: set-up, data integration and system's life cycle. In the 'set-up' phase, several existing ontologies are integrated and merged: the Gene Ontology, the Relations Ontology, the Molecular Interactions ontology, an upper level ontology (see 'An upper level ontology for application ontologies in the life sciences' section) and an ontology holding taxonomical terms for the four model organisms supported by CCO (*A. thaliana*, *H. sapiens*, *S. cerevisiae *and *S. pombe*). A core cell cycle ontology is generated as output from this set-up phase, which in turn serves as input for the 'data integration phase' where GOA annotations and protein data, such as protein-protein interactions, are integrated. Finally, 'life cycle' phase depicts the maintenance of the system by considering the stages of updating the integrated data (such as ontologies, protein data). This phase also shows the generation (export) in different formats for further exploitation.

CCO is built from scratch every three months, and only the identifiers are kept for consistency between releases. This automatic pipeline encompasses the typical life cycle of an integrated system: set-up, data integration and system maintenance. All the integrated information is cross-referenced to the original sources to ensure data provenance. The integration pipeline relies on the ability to programmatically manipulate ontologies, terms and relations, a functionality offered by ONTO-PERL [57]. The code of the pipeline is available as supplementary material [119]. The output of the pipeline is four species-specific ontologies, plus a composite ontology that integrates the species-specific ontologies via orthology relationships. Figure 6 depicts the system integration pipeline and the various phases of its development.

#### Set-up

In this initial phase, the ontology structure and its lexicon (for formal ontology definitions, see [102]) are created. The core CCO ontology is built from the upper level ontology (ULO; see Materials and methods) and the latest releases of GO, RO, Molecular Interactions ontology (MI) [103] and NCBI taxonomy in the order specified. Initially, a 'pre-cell cycle ontology' is built that constitutes the backbone for the CCO ontologies (the four species-specific ontologies and the integrated ontology). The main data source used for CCO is GO. From the 'biological processes' subontology of GO, the complete branches under the terms 'cell cycle' (GO:0007049), 'cell division' (GO:0051301), 'cell proliferation' (GO:0008283), 'DNA replication' (GO:0006260) and 'chromosome segregation' (GO:0007059) with all their descendants are imported ('pre-cell cycle ontology'). These five branches are linked as children to the term 'cellular process' (GO:0009987), which in turn becomes a child of the term 'biological process' (GO:0008150). We refer to the terms contained in these branches as 'cell cycle' terms. Currently, 3,295 cell cycle biological process terms are imported from the GO. In addition, the entire 'cellular component' and 'molecular function' subontologies are imported.

Every entity within CCO is given a unique identifier of the form CCO:Xnnnnnnn where 'CCO' represents the ontology namespace, 'X' the entity subnamespace, and 'nnnnnnn' a sequence of seven digits. The legal subnamespaces include: 'C' for cellular components, 'P' for biological processes, 'F' for molecular functions, 'B' for proteins, 'G' for genes, 'T' for taxon terms, 'I' for interactions, 'O' for orthology terms, 'Y' for interaction types and 'U' for ULO terms. All the original identifiers of the imported terms are stored in the 'xref' section. Additionally, an association table keeps track of the mapping between GO identifiers and CCO identifiers. Next, the RO is fully incorporated and the 'interaction type' branch from the MI is integrated and then a specific taxonomy is built based on the NCBI taxonomy for *H. sapiens*, *S. cerevisiae*, *S. pombe *and *A. thaliana*.

#### Data integration

Data integration follows a protein centric model in which the representations of proteins are considered as integration pivots that connect their relevant data (such as the molecular functions in which they participate).

We define the proteins annotated with cell cycle terms (as defined in the section 'Set-up' above) in the corresponding GOA files [104] as the 'core cell cycle proteins'. These proteins are added to CCO as the children of the term 'core cell cycle protein' (CCO:B0000000) and used as the starting point (seed) for the data integration process. Currently, CCO has 1,648 'core cell cycle' proteins for *S. cerevisiae*, 3,276 for *A. thaliana*, 1,348 for *S. pombe *and 9,114 for *H. sapiens *(Table 2).

The 'core cell cycle' protein section of CCO is then expanded to include the proteins known to interact with the core cell cycle proteins, as documented in IntAct (only one degree of separation). The protein role (prey, bait or neutral component), type of experiment (such as yeast two hybrid), type of interaction (such as physical association), and so on are all retained in CCO. Then, protein information (such as synonym names, encoding genes and cross-references) are retrieved from the UniProt knowledge base. In addition, post-translational modification data, when available in UniProt, are also added by creating new terms defined by their specific modification. Finally, the OrthoMCL clustering utility is used to generate clusters of putative orthologs for the four species included in the CCO. The parameters used for running OrthoMCL have been chosen to obtain a balanced size of the resulting clusters. Compared to the default values, the following parameters were changed to make the clusters more homogeneous: *pv_cutoff *= 1e-6, *pi_cutoff *= 25, *inflation *= 4. The input all-against-all BLAST matrix for the four complete proteomes is produced with the Tera-BLAST hardware implementation of BLAST [105] with default parameter settings. All the proteins classified by OrthoMCL as orthologous to the core cell cycle proteins are incorporated (together with their corresponding genes and other protein information) into the integrated CCO ontology (Table 3). In this way, the orthology data glued the four specific ontologies together by adding the cluster types to which the proteins of the different species belong. All the imported entries from the sources are cross-referenced via 'xref' tags so that the data can be tracked back to their sources. Finally, the four organism-specific ontologies and the CCO composite ontology (output of this phase) are checked and made available, effectively producing the official release of the system.

All the entities in CCO (proteins, including their modified forms, genes, interactions, and so on) are modeled as classes (also known as universals [106]) since they gather shared commonalities that are present in all the particulars (instances) they represent. Some of the formal ontology design principles as specified by OBOF have been relaxed while building CCO. For instance, it is evident that the simple fact represented by a relationship between a given protein P and a particular location L ('P located in L') can be read as: 'all proteins of type P are located in L' or 'some of proteins of type P are located in L'. Actually, the best interpretation of GOA annotations could be 'some of P may be located in L'. What the case is depends on a particular cell type, particular timing (in the development or cell cycle) and so on. Therefore, such statements should be considered carefully while performing the analysis [107,108].

#### System maintenance

Semantic improvements are incorporated in this phase, further enriching the ontology (see section "Improving the OWL version of the Cell Cycle Ontology). Validation and verification processes are carried out while building CCO to ensure its soundness. The ontologies generated in the previous phase are manually and automatically checked by ontology editors, validators and reasoners. In addition, the pipeline log execution files are inspected in detail. These files are sufficiently detailed so as to point out any possible problems. This has allowed us to assemble a fully automated pipeline that uploads the ontologies and their exports in the different formats to the CCO website and to all related and supporting repositories: CCO Website [109]; BioPortal [52]; Ontology Lookup Service [110]; BioGateway [111]; CCO Subversion (SVN) repository [112]; CCO Concurrent Versions System (CVS) repository [113].

For each release, the entire set of sources (ontologies, databases, and so on) is retrieved from their repositories. Therefore, CCO is a dynamic artifact in which the terms and relationships are updated as new data are released. Versioning servers keep track of all the changes between different releases.

#### An upper level ontology for application ontologies in the life sciences

**Figure 7:**
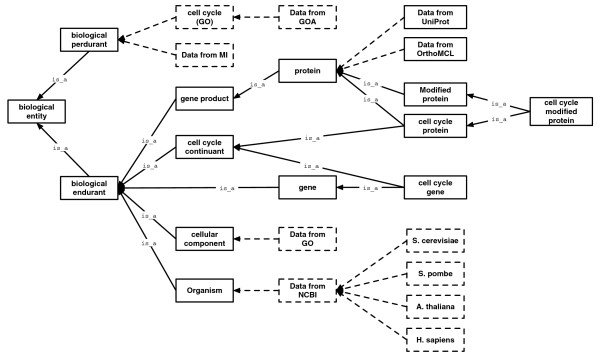
Upper level ontology for CCO. The ULO provides a hierarchical scaffold, including generic terms (for example, cell cycle gene), which serve as 'hooks' for hanging the integrated resources. The dashed rectangles represent the type of data residing below the parental nodes: the node called 'Data from GO' under the term 'cellular component' shows the placeholder where the cellular components from GO are placed (for example, nucleus), the node 'Data from UniProt' under the term 'protein' shows the placeholder where protein data from UniProt resides (for example, p53), and so forth.

A ULO is an ontology that structures very general types of concepts (such as a process) in generic as well as specific domains [114] to provide an integration scaffold for including other ontologies. A ULO connects a relatively small number of concepts by meaningful and strictly defined relationships. To accommodate the natural interlinkage of terms and relationships for cell cycle knowledge, we developed a ULO for CCO (Figure 7). The implementation of this ULO was based on some of the concepts presented in the Basic Formal Ontology [115,116] to ensure the interoperability of CCO with other ontologies. Our ULO has been customized for CCO by the inclusion of a few high-level terms such as 'cell cycle gene'. The developed ULO is generic and can also serve other subdomains of life sciences (for example, programmed cell death) with minor modifications.

#### Improving the OWL version of the Cell Cycle Ontology

The Ontology Pre-Processor Language (OPPL) [117,118] is a language for manipulating OWL ontologies. OPPL is based on the Manchester OWL syntax, and is used to write macros to be applied to an OWL ontology. The OPPL macros are written by the user in a flat file that is processed by the OPPL program, which executes the instructions and generates a new ontology. OPPL macros can be used to add or remove entities and axioms (for instance, 'subClassOf part of some all (participates_in only (process and interaction))'). In the example below, a sample definition of axioms or transformations on the CCO are made with OPPL (statements end with semicolon and comments (not processed) start with hash):

# Add a class called 'interaction'.

# Add the following necessary condition to the newly added

# 'interaction'class, the participants are only the union of

# protein_1 and protein_2.

# Add the rdfs:label 'interaction' to the newly added

# 'interaction' class.

ADD Class interaction;

ADD subClassOf has_participant only (protein_1 or protein_2);

ADD label 'interaction';

# Select any class that has the following condition as a

# superclass: the participants are only the union of

# protein_1 and protein_2.

# Remove the rdfs:label 'interaction' from any selected class.

# Add the rdfs:label 'interaction of protein_1 and protein_2'

# to any selected class.

SELECT subClassOf has_participant only (protein_1 or protein_2);

REMOVE label 'interaction';

ADD label 'interaction of protein_1 and protein_2';

OPPL is used to enrich the OWL ontology that the CCO pipeline builds from the original OBOF files. The advantages of OPPL are automatic maintenance and consistent, explicit and flexible development. OPPL also has the capability of performing what would otherwise be prohibitive using a graphical interface. The set of axioms used to enrich CCO via OPPL can be found in [119].

OPPL has also been used to apply ontology design patterns (ODPs) in CCO [120,121]. ODPs are ready-made modeling solutions for ontologies, thoroughly tested and documented, and offer an abstraction to be reused in different implementations. The abstraction also means that a developer need not know all the modeling details, and can use the ODPs as self-sufficient modeling components. Within CCO, two ODPs were used: the Sequence ODP [122] and the ULO ODP [123]. The reasoning was applied to the fragments of CCO ontologies that contained only the 'cell cycle' branch of GO (and the terms associated with this branch) because the size of the complete ontologies was prohibitive for the deployment of currently available reasoners.

## Availability

All the software and data of the CCO project are freely available, either upon request or through websites [112,113] (both through CVS and SVN). An interactive SPARQL query interface is available at [73]. CCO can also be browsed, searched and visualized through the BioPortal at [52]. CCO is also available at The Ontology Lookup Service [110]. Comments and suggestions about CCO can be exchanged through a mailing list ccofriends@psb.ugent.be.

## Abbreviations

CCO: Cell Cycle Ontology; CVS: Concurrent Versions System; DL: Description Logics; GML: Graph Modeling Language; GO: Gene Ontology; GOA: Gene Ontology Annotation; MI: Molecular Interactions ontology; NCBI: National Center for Biotechnology Information; OBO: Open Biomedical Ontology; OBOF: Open Biomedical Ontology Format; ODP: ontology design pattern; OPPL: Ontology Pre-Processor Language; OWA: Open World Assumption; OWL: Web Ontology Language; RDF: Resource Description Framework; RO: Relation Ontology; SVN: Subversion; ULO: upper level ontology; URI: Uniform Resource Identifier; XML: eXtensible Markup Language.

## Authors' contributions

EA was the main architect and engineer of CCO. ME contributed expertise in bio-ontologies, ODPs and OPPL. RS provided bio-ontology expertise. WB built sample SPARQL queries and developed the transitive closures. AI and IB developed the CCO visualizer. VM contributed cell cycle expertise, ontology design and platform engineering skills. BDB and MK contributed their expertise about knowledge management in systems biology and led the project. All authors contributed to the writing of the manuscript.
